# The Influence of Microglia on Neuroplasticity and Long-Term Cognitive Sequelae in Long COVID: Impacts on Brain Development and Beyond

**DOI:** 10.3390/ijms25073819

**Published:** 2024-03-29

**Authors:** Luana da Silva Chagas, Claudio Alberto Serfaty

**Affiliations:** 1 Program of Neuroscience, Department of Neurobiology, Institute of Biology, Federal Fluminense University, Niterói 24210-201, Rio de Janeiro, Brazil; luana_chagas@id.uff.br; 2 National Institute of Science and Technology on Neuroimmunomodulation—INCT-NIM, Oswaldo Cruz Institute, Oswaldo Cruz Foundation, Rio de Janeiro 21041-250, Rio de Janeiro, Brazil; 3 Rio de Janeiro Research Network on Neuroinflammation, Oswaldo Cruz Institute, Oswaldo Cruz Foundation, Rio de Janeiro 21041-250, Rio de Janeiro, Brazil

**Keywords:** neuroinflammation, neuroplasticity, microglial function, critical periods, neurodegenerative diseases, SARS-CoV-2

## Abstract

Microglial cells, the immune cells of the central nervous system, are key elements regulating brain development and brain health. These cells are fully responsive to stressors, microenvironmental alterations and are actively involved in the construction of neural circuits in children and the ability to undergo full experience-dependent plasticity in adults. Since neuroinflammation is a known key element in the pathogenesis of COVID-19, one might expect the dysregulation of microglial function to severely impact both functional and structural plasticity, leading to the cognitive sequelae that appear in the pathogenesis of Long COVID. Therefore, understanding this complex scenario is mandatory for establishing the possible molecular mechanisms related to these symptoms. In the present review, we will discuss Long COVID and its association with reduced levels of BDNF, altered crosstalk between circulating immune cells and microglia, increased levels of inflammasomes, cytokines and chemokines, as well as the alterations in signaling pathways that impact neural synaptic remodeling and plasticity, such as fractalkines, the complement system, the expression of SIRPα and CD47 molecules and altered matrix remodeling. Together, these complex mechanisms may help us understand consequences of Long COVID for brain development and its association with altered brain plasticity, impacting learning disabilities, neurodevelopmental disorders, as well as cognitive decline in adults.

## 1. Introduction

Neuroinflammation is mediated by reactive microglia and astrocytes in response to peripheral immune cell trafficking and plays a significant role in pathophysiological mechanisms of the acute phase of COVID-19 [1,2]. While astrocytes, but not microglia, are identified as the primary target and viral reservoir during SARS-CoV-2 infection [3], disruptions in cerebral homeostasis, including the potential of vascular damage, can alter microglial functional properties. Microglia dysregulation has implications for development and neuroplasticity across different life stages, including critical periods of neural circuitry development and the maintenance of healthy synapses in adults (reviewed in [4]. Understanding the neural damage induced by viral infections during the acute phase is crucial for unraveling the mechanisms behind ‘Long COVID’ that lead to abnormal conditions including cognitive and neuropsychiatric disorders.

Long COVID, also termed Post-Acute Sequelae of COVID-19 (PASC), is a multisystem syndrome that encompasses a variety of new, recurrent or continuous symptomatology that persist after an acute SARS-CoV-2 infection. Neurological symptoms may vary in duration, from 30 days up to weeks or several months without an alternative diagnosis [5,6,7,8]. Approximately 80% of hospitalized COVID-19 patients exhibit neurological symptoms, correlating with an increased risk of mortality. The extent of this association with late neurological manifestations remains unclear [9].

Long-term neurological complications have been seen in other respiratory infections, such as in cases of severe acute respiratory syndrome coronavirus (SARS) [10] and Middle East respiratory syndrome coronavirus (MERS) [11]. A 4-year follow-up study revealed that, despite physical recovery, certain psychiatric conditions, such as PTSD, depression, panic disorder and obsessive–compulsive disorder, along with chronic fatigue, persisted in SARS infection [12]. Furthermore, to this day, there is still no clear evidence regarding the cause of post-encephalitic parkinsonism symptoms emerging from the lethargic encephalitis (“the sleepy sickness”) that affected over one million people during the 1918 Spanish flu pandemic caused by influenza A (H1N1) [13,14]. More recently in history, a similar condition of parkinsonism was also reported in survivors of avian flu [15], and, more recently, of COVID-19 [16,17,18]. Despite studies being limited to raising evidence of a direct causal association, there is a clear need for more robust research on the matter. 

A recent cohort assessed the increased risk of neurological, cognitive and mental health complications up to 1-year post-acute COVID-19, even in non-hospitalized patients. These included cerebrovascular issues, cognitive disorders like brain fog and Alzheimer’s disease, drowsiness, peripheral nervous system disorders, migraines, seizures, sensory alterations (persistent anosmia and/or ageusia, tinnitus, vision impairments), musculoskeletal disorders, Guillain–Barré syndrome, encephalitis, anxiety, depression, psychotic disorders and PTSD [19,20]. Such complications have been associated with signs of neurodegeneration [21], neuroinflammation [22], and demyelination [23], but little is discussed about the impairments associated with the abnormal neuroplasticity mechanisms that may be also involved.

In children, late manifestations of SARS-CoV-2 and its variants can induce symptoms such as headaches, fatigue, palpitations, anosmia, difficulty concentrating and insomnia. These symptoms may persist even in silent or mild cases of an acute COVID-19 infection [24], and may contribute to the neglect of potential risks of sequelae, particularly neurological/psychiatric sequelae, associated with Long COVID. Besides the nonspecific viral symptoms, the increased vulnerability to stressors induced by the pandemic (undernutrition, social isolation, sick family members) during the early stages of brain development may worsen cases of pediatric Long COVID, amplifying neuropsychiatric impacts [25]. Hence, establishing the prevalence of pediatric ‘Long COVID’ is essential for assessing risks related to delays in childhood neurodevelopment, academic performance and potential long-term medical and neuropsychiatric outcomes.

The present non-systematic review intends to discuss the central role of microglia in orchestrating the long-term outcomes resulting from the acute phase of COVID-19, focusing on mechanisms associated with neuroplasticity and abnormal synaptic pruning. These impairments may pose a greater risk and increase vulnerability to the development of neurological and neuropsychiatric symptoms of Long COVID, especially during childhood. We will also review the key mechanisms involved in microglial-dependent neuroplasticity that might be useful for establishing strategies for the rehabilitation of Long COVID symptoms.

## 2. Neuroinflammation Mechanism during the Pathogenesis of COVID-19

During COVID-19 pathogenesis, much of the mechanisms involved in neuroinflammation result from systemic inflammation that blunts monoamine neurotransmission, decreases trophic factors and activates both astrocytes and microglia. Beyond the phagocytosis of damaged cells, activated glia release inflammatory mediators result in an excess of glutamate, the activation of the quinolinic acid pathway, pro-inflammatory cytokines expression, such as TNF-α and interleukins, and abnormal complement cascade activation [26,27]. Elevated levels of glutamate and NMDA receptors impact neuronal activity and excitotoxicity, potentially causing alterations in learning, memory and neuroplasticity, along with hallucinations and nightmares that could exacerbate or establish a new onset of Long COVID neurological symptoms (reviewed in [28,29]).

In the case of COVID-19, the pathophysiological process is initiated by three main factors inherent to this disease: peripheral cytokine storm, vascular hyperpermeability and the dysregulation of the renin–angiotensin system in the CNS (discussed in [30]. In summary, the pathobiological process begins with the virus reaching the ACE-2 enzyme receptors that are widely distributed in all organs, including neural tissue. The downregulation of ACE2 and recognition of the viral Spike glycoprotein by cells of the innate immune system establish local inflammation through the cytokine storm, and this varies according to the viral entry route (respiratory, gastrointestinal, etc.). This, in turn, is amplified by other immune cells (neutrophils, effector T cells, monocytes and macrophages), adding cytokines such as interleukin-6 (IL-6), interleukin-1b (IL-1β) and tumor necrosis factor-alpha (TNF-α) to the inflammatory process [31,32].

Among the two debated routes of SARS-CoV-2 entry into the brain, we have the hematogenous route [33], including a transcellular pathway by which SARS-CoV-2 can cross the BBB [34], and the axonal transport from olfactory sensory neurons [35]. In an attempt to investigate the cause of anosmia, researchers failed to find evidence of viral infection in these neurons. Clinical studies revealed that support cells, not neurons, are the primary targets of SARS-CoV-2 in the upper airways, based on post-mortem tissue analyses of individuals who died from COVID-19 complications [36]. 

Nevertheless, anatomically, the olfactory bulb could be a convenient route for the virus to reach the hippocampus, a structure associated with cognitive processing, learning and short-term memory, which potentially justifies the possibility of an accelerated cognitive decline in individuals who are more susceptible to respiratory infections [37]. For instance, studies in hamsters that analyzed the impact of SARS-CoV-2 infection on the olfactory pathway observed an incomplete recovery of sensory neurons, prolonged glial activation in the olfactory bulb and a reduced dendritic spine density in the hippocampus after the acute phase of infection, supporting the long-lasting olfactory and cognitive effects observed in Long COVID [38]. 

Despite the existence of a selective blood–brain barrier (BBB), the central nervous system (CNS) is in constant interaction with the peripheral immune system through the choroid plexus and circumventricular organs/regions, which are also possible entry routes for the virus into the CNS, along with the olfactory epithelium and the BBB itself, damaged by systemic inflammation (reviewed in [30]. BBB damage is correlated with the neurological sequelae of COVID-19 due to the downregulation of endothelial ACE2 receptors. BBB disruption, in association with hyperinflammation, results in endothelins that promote the extravasation of pro-inflammatory cells and activate cytokine cascades and complement activation in the brain parenchyma, contributing to vascular fragility and a hypercoagulable state [39,40,41]. 

The association between neuroinflammation and the neurological sequelae of Long COVID has been supported by the work of Yang and colleagues, demonstrating that subpopulations of microglia and astrocytes associated with COVID-19 share characteristics with pathological states previously observed in human neurodegenerative diseases [2,42,43]. The molecular disruption related to COVID-19 overlaps with those found in chronic neurological diseases, where genetic variants associated with cognitive decline, schizophrenia and depression reside [2]. A longitudinal study detected that certain cytokines, such as IL-1β, IL-4 and IL-6, and markers of neuronal dysfunction, such as beta-amyloid protein, total tau, p-T181-tau, light chain neurofilament and neurogranin, a marker of neuronal extracellular vesicles, remain elevated in the plasma of patients recovering from COVID-19 who self-reported neurological issues around 1 to 3 months after the initial infection [44]. TNF-α has also been shown with increased plasma levels in Long COVID patients [45]. This suggests that the neuroinflammatory processes during initial SARS-CoV-2 virus infection induce sustained reprogramming in the phenotype of CNS cells contributing to peripheral inflammation, neuroinflammation, neurodegeneration and persistent systemic effects after COVID-19 and determine the neurological symptoms observed in Long COVID.

Besides this, much evidence indicates that human neurons are less susceptible to SARS-CoV-2 infection compared to astrocytes, recognized as a primary target for secondary CNS infection and a viral reservoir [3,46]. While microglia are key players in inflammatory processes in neurological diseases [26], there is limited evidence of direct SARS-CoV-2 infection in these cells. 

Nevertheless, microglia remain crucial in the broader context of neuroinflammatory responses in Long COVID, alongside neuroimmune and neuroplastic reactions. For instance, the NLRP3 inflammasome, implicated in neuroinflammation, modulates neuroplasticity. Studies in mice show that the pharmacological inhibition of the NLRP3 inflammasome prevents synaptic failure and promotes long-term potentiation (LTP) [47]. Moreover, its inactivation also promotes antidepressant effects by neuroplasticity enhancement [48]. Preclinical evidence suggests that SARS-CoV-2 activates the NLRP3 inflammasome, potentially contributing to the emergence or persistence of neurological and psychiatric symptoms through altered neuroplastic mechanisms [49]. Additionally, the elevated ATP levels induced by SARS-CoV-2 may also stimulate the NLRP3 inflammasome through P2X7 receptor hyperactivation, primarily expressed in microglia and astrocytes and associated with neuroinvasive and neuroinflammatory processes in psychiatric and neurodegenerative diseases [50]. Therefore, P2X7 and NLRP3 present promising therapeutic avenues to be explored for the treatment of neurological complications in COVID-19 patients.

## 3. Microglial and Neuroplasticity: Impact on Neurological Outcomes of Long COVID

Microglia play a fundamental role in CNS plasticity. They are actively enrolled in synaptic selection and the activity-dependent reorganization of neural circuits throughout development [4]. Among various physiological functions, microglia promote the formation [51], maturation [52] and selective elimination of immature synapses [53], a basic requirement for proper brain development. Microglia are actively involved in neuroplasticity; they have been shown to regulate extracellular matrix remodeling [54] and to be necessary for use-dependent cortical plasticity [55,56].

As the CNS matures, microglia monitor any threats to brain homeostasis through their “sensome” and processes and are capable of sensing molecular changes in the microenvironment and neuronal activity [57,58]. When the environment challenges the nervous system (e.g., brain injury, infection, alcohol exposure or dietary restrictions), microglia transit between dynamic reactive phenotypes and modify their density, morphology and molecular signature, which results in the proper tuning of their function [59]. For instance, early malnutrition [60], early life stress or maternal immune activation alter the microglial role in synaptic pruning and, thus, plasticity, impacting social behavior [61] and sensory neural processing [62]. 

Thus, as a primary strategy for homeostasis maintenance, microglia permanently assume the role of making any necessary adjustments for the construction or restoration of neural function. In environmental CNS imbalance, microglia respond through an extensive system of molecular recognition (chemokines, cytokines, trophic factors) and interact bidirectionally with neural and immune cells to influence their response. Therefore, microglial dysfunction, marked by a loss of physiological functions or an excessive inflammatory response, implies a higher risk of neurodevelopmental disorders such as autism and schizophrenia [4].

In humans, a comprehensive longitudinal study using the UK Biobank dataset detected significant gray matter loss within approximately 6 months between imaging sessions, with a reduction in the thickness and cortical volume of the left hemisphere [63]. This loss occurred in areas such as the anterior parahippocampal gyrus (episodic memory), lateral orbitofrontal cortex (secondary olfactory cortex) and superior insula (anxiety behavior). Additionally, when hospitalization was considered as a variable, alterations were detected in areas associated with smell, emotion, memory and learning, such as the left cingulate cortex, right hippocampus and right amygdala, related to anxiety behavior. Interestingly, most of the participants experienced only mild symptoms of COVID-19, even though the authors detected an accelerated reduction in whole-brain volume and more pronounced cognitive declines associated with heightened atrophy of a cognitive lobule in the cerebellum (crus II), among other long-lasting deleterious effects on brain structure and function, when compared to the control group [63]. This study provides a distinctive perspective on COVID-19-related changes in the brain structure, shedding light on the interplay between neuroplasticity and the potential for neurocognitive outcomes. 

In the adult brain, microglia play a crucial role in long-term plasticity and neurogenesis and are essential for learning and memory formation. Traditional neurogenic sites, like the olfactory bulb and the hippocampal dentate gyrus, continuously generate new neurons throughout life [64]. The microglia function on this matter was demonstrated when the microglial depletion led to less functional neurons in the olfactory bulb, affecting olfactory responsiveness [65]. The proper integration of new neurons into hippocampal circuits, both structurally and functionally, also relies on microglial function [66]. In homeostatic conditions, microglia interact with synapses, synchronizing local neuronal activity and influencing behavioral and cognitive functions [67]. These processes are regulated by the physiological production of inflammatory cytokines, such as interleukins and TNF-α, which contribute to adult neurogenesis and efficient axonal plasticity in response to peripheral inflammation [68,69]. 

Another crucial player under investigation, due its role in neuronal regulation, survival and neural plasticity, is the brain-derived neurotrophic factor (BDNF). Reductions in plasma and brain BDNF levels are common in patients with psychiatric and neurodegenerative diseases, possibly secondary to a state of chronic inflammation affecting the brain [70]. Considering that BDNF is downstream in the ACE2-Mas axis [71], the current literature discusses the potential of the SARS-CoV-2-ACE2 interaction to reduce its levels, with implications for the pathogenesis of Neuro-COVID [72]. Indeed, microglial BDNF is essential for motor learning improvement in structural, behavioral and electrophysiological impairments in animals with the conditioned depletion of this molecule [73]. Besides this, Gonzalez and colleagues have previously shown that the intrahippocampal stimulation of the immune system with pro-inflammatory cytokines decreases BDNF expression and potentially compromises the brain’s neuroplastic functions, such as neurogenesis, LTP and dendritic sprouting [74]. Moreover, longitudinal studies to delve into the investigation of the association between trophic support that imbalances and structural changes leading to neurological or neuropsychiatric manifestations in Long COVID patients are scarce. Recently, a meta-analysis assessed relevant studies that identified lower BDNF levels in patients with COVID-19 when compared to healthy controls; this dysregulation was also related to the severity of the disease and reported in a unique study that assessed Long COVID patients, possibly linking this mechanism to neurological outcomes [75]. 

Despite the importance of microglia in CNS homeostasis, other immune cells also seem to contribute to its physiological role [76]. In addition to the immune response and neural activity, memory and learning tasks and social behavior are modulated by the secretion of cytokines by CD4+ T lymphocytes in the meningeal spaces, the latter being associated with IFN-γ [77]. The release of IFN-γ by T lymphocytes is also implicated in synaptic elimination by microglia and cognitive sequelae, as illustrated by spatial learning deficits in an animal model of flavivirus infection [78]. Also, a subset of innate lymphocytes is involved in cortical inhibitory synapse formation with impacts on social behavior development [79]. This may have important implications in the recent context of COVID-19, where the mechanisms associated with the long-term cognitive sequelae of Long COVID are still under investigation.

## 4. Molecular Signals That Regulate Microglia-Dependent Neuroplasticity

Molecular cues, identified as “find-me”, eat-me” and “do not eat me” signals, regulate the proper guidance of microglia/macrophages towards phagocytosis, which is relevant for sculpting neural connections during development and, more recently, has been implicated in many neurocognitive and neuropsychiatric conditions (reviewed in [80]. 

In different CNS areas, it has been well established that synaptic pruning mediated by microglia is complement-dependent during postnatal development [81,82,83], with microglia being responsive to the fractalkine primarily released by neurons and endothelium [84]. Evidence in the adult brain indicates how fractalkine-mediated neuroplasticity can modulate cognitive function. The absence of Cx3CR1, the fractalkine receptor in microglia, impacts neurogenesis, weakening learning tasks [83,85]. Abnormal levels of TGF-β or CX3CR1 have also been shown to result in aberrant neuroplasticity in adulthood [85,86,87]. The immune role in cognitive function has been demonstrated in CX3CR1 KO animals, where microglia’s function in hippocampal synaptic maturation was analyzed, showing significant delays associated with the number of microglia. Subsequent studies revealed alterations in social interaction and neural connectivity in adulthood [83,88,89]. 

In 2020, Scott-Hewitt and colleagues showed in vivo that the synaptic exposure of phosphatidylserine (PS) in the hippocampus and retinogeniculate areas assumes a temporal dynamics correspondence with synaptic pruning, while C1q-deficient mice failed to properly refine retinogeniculate connections, suggesting a functional interaction between the complement system and PS-mediated synaptic pruning [90]. There are increasing studies in the literature assessing the implications of C1q as an early driver of synaptic loss in neurodegenerative and neuroinflammatory conditions [91,92] (reviewed in [93]). Microglial depletion or the inhibition of its phagocytic function prevents forgetting, dissociation of engram cells and complement-mediated synaptic loss, allowing mice to forget non-essential environmental cues [94]. Deficits in hippocampal neurotransmission, as well as cognitive and behavioral impairments, are also associated with a lack of C1q in adulthood [95]. Like SARS-CoV-2, residual cognitive sequelae are also present in other viruses. The West Nile virus (WNV), a neurotropic RNA virus like SARS-CoV-2, can promote the elimination of presynaptic terminals after the viral infection of adult hippocampal neurons in a complement-dependent manner, and it is associated with memory dysfunction in human studies and animal models [27].

On the other hand, CD47 signaling and its SIRPα receptor constitute a well-known example of a “don’t eat-me” signal that prevents aberrant microglia-mediated phagocytosis. CD47 is localized in more active synapses, and disruptions to either CD47 or SIRPα in knockout studies increased synaptic over-pruning in the retinogeniculate system during early development [96]. Intriguingly, it appears that the proteolytic cleavage of SIRPα by metalloproteinases (MMP) in response to neuronal activity releases an extracellular SIRPα domain, which binds to presynaptic CD47 and promotes the maturation of presynaptic terminals [97]. The regulatory role of SIRPα in synaptic pruning was evident in a model of Alzheimer’s Disease, where the authors observed that the loss of microglial SIRPα increased synaptic loss mediated by microglia engulfment and enhanced cognitive impairment, while, in human tissue, microglial SIRPα expression declines alongside the progression of Alzheimer’s disease [98]. Indeed, synaptic elimination mechanisms in adults, as evidenced by microglial dysfunction in synaptic pruning, have been suggested in studies using post-mortem tissue from COVID-19 patients [99] and neuroimaging studies showing signs of synaptic loss [63].

Although the role of neuronal activity and microglia in synaptic refinement is well established [82,100], we still do not know exactly how these two distinct mechanisms are linked and how neural activity can be translated into local cues mediating the microglial engulfment of synaptic elements. Most of the complement-based studies on Neuro COVID or Long COVID studies have, so far, focused on these molecular signals as mechanisms of virus resistance [101] and on the worsening of coagulopathies that enhances the risk of Long COVID [102]. Up to this point, these findings underscore the need for a deeper understanding of how viral infections can impact molecular aspects of microglial function on synaptic selection and influence neural plasticity as an underlying cause in the neurological symptoms found in Long COVID.

## 5. Critical Periods of Brain Development and the Risk of Long COVID in Children

The infant brain is under rapid development and is highly susceptible to environmental stressors. Understanding the pathophysiological mechanisms behind the risks associated with the acute effects of COVID-19 on the developing brain during critical periods has been debated elsewhere [59], but little is known about the long-term cognitive, behavioral and psychiatric implications related to Long COVID in children and adolescents. Besides this, COVID-19 frequently presents mild acute clinical manifestations in children, which could possibly mask the potential risks of sequelae and raise appropriate concerns for public health.

Considering that microglia actively participate in brain plasticity by sculpting neural circuits and selecting and eliminating synapses, inappropriate networks may arise as a consequence of abnormal microglial performance during critical periods of development. If not detected or reversed within this sensitive developmental window, this can lead to long-term neurodevelopmental and psychiatric disorders [103]. A preliminary case–control study in young patients detected a significant increase in microgliosis in younger COVID-19 patients compared to older ones [104]. This suggests a potentially primed, more reactive microglia response in younger patients who, despite exhibiting mild acute symptoms, might be prone to the onset of Long COVID sequelae.

Recently, a study using human organoids provided evidence that SARS-CoV-2 infection may lead to excessive, disordered and premature synaptic elimination during the course of the disease, similar to what is observed in other neurological disorders such as schizophrenia, Alzheimer’s disease and Parkinson’s disease [105]. The disruption of neural circuitry integrity through an abnormal microglia-mediated synaptic over-pruning emerges as a potential mechanism in the onset of neurological, cognitive and psychiatric symptoms in COVID-19 recovery patients. 

To investigate the impact of immune activation and microglial priming on vulnerabilities to neurodevelopmental disorders, microglial-like cells with a pro-inflammatory phenotype were successfully generated from mononuclear cells derived from the umbilical cord of both exposed and unexposed mothers [106]. This approach validates the search for strategies that could help to identify neonates and children with vulnerabilities caused by SARS-CoV-2, among other viral infections. Besides this, this highlights the importance of microglial function and the risks posed by its priming in neural development and subsequent disease emergence later in life.

Regarding synaptic maturation, at the end of critical periods, the perineuronal networks (PNNs), specialized reticular formations of the compact extracellular matrix that envelop neuronal subsets and stabilize proximal synapses, can be modified by microglia sculpting [107]. Furthermore, it has been shown that microglial depletion induces increased cortical perineuronal nets and heightened neural activity in excitatory cortical neurons [108]. Also, it has been demonstrated that MMP-9 contributes to the stabilization of synapses during critical periods of development and the inhibition of MMP-9 activity in vivo resulted in an altered topographical refinement of retinocollicular connections [109]. Therefore, the dysregulation of extracellular matrix dynamics due to neuroinflammation and microglial dysfunction may disrupt critical periods and neural circuit formation, posing a risk for cognitive and psychiatric sequelae following viral infection.

Also, it has been shown in the rodent visual system that synaptic plasticity and circuitry responses to environmental modifications depend on microglial functional integrity [56]. Therefore, microglial homeostasis is a crucial issue during brain development, when synaptic adjustments are necessary for the correct processing of neural information. Since microglial homeostasis can be disrupted not only by viral infections but also by exposure to nutritional stress, alcohol consumption during pregnancy, environmental stress and lack of appropriate care during infancy, ensuring plain conditions of neural development during the first years of life seems to be necessary for the full development of cognitive functions [4].

**Figure 1:**
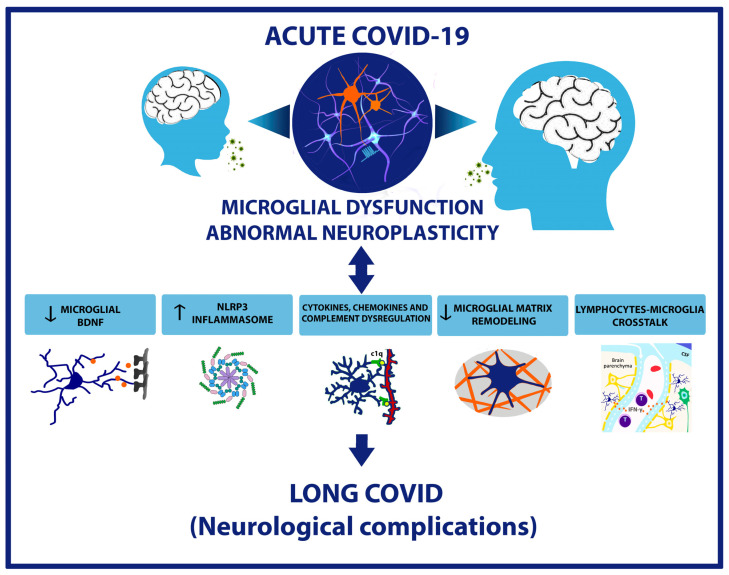
Neuroplasticity impairments due to microglial dysfunction as a central mechanism for “Long COVID” neurological outcomes. Various mechanisms, including neuroimmune interactions, lead to microglial dysfunction, which directly impairs neural functions, reproducing “Long COVID” neurological and cognitive symptoms. Some mechanisms related to microglial regulation are being explored, such as the decrease in microglial BDNF levels and involvement of NLRP3 inflammasome, linked to the decrease in LTP; the dysregulated expression of cytokines, chemokines and complement system molecules that interfere with proper synaptic plasticity and induce insufficient or excessive synaptic stripping; the lack of microglia influence on matrix remodeling that promotes E/I imbalance and the impairment of neural circuit maturation; and the cross-talk between microglia and other circulating immune cells, like CD4+ T lymphocytes, which further exacerbates these effects. These mechanisms illustrate how abnormal plasticity contributes to Long COVID neurological complications, underscoring the importance of targeting them for therapeutic strategies to improve neuroplasticity.

In conclusion, neuroplasticity alterations mediated by microglial dysfunction and abnormal synaptic pruning appear as key elements in Long COVID syndrome, affecting both the developing and the adult brain (Figure 1). Therefore, future research should address strategies to reestablish appropriate synaptic plasticity, including the comprehension of molecular mechanisms involved in abnormal synaptic pruning, the signaling involved in the interaction between the complement system and the expression of molecular signals in synaptic selection as important issues for the resolution of cognitive deficits following Long COVID syndrome found in infants and adults. While Long COVID causes important but frequently time-limited alterations in adults, it is noteworthy that SARS-CoV-2 infections in children may result in possible life alterations in neural circuitry development and plasticity, impacting learning abilities and socialization throughout life. 

## Data Availability

Not applicable.
